# The mitochondrial amidoxime reducing component—from prodrug-activation mechanism to drug-metabolizing enzyme and onward to drug target

**DOI:** 10.1016/j.jbc.2023.105306

**Published:** 2023-09-29

**Authors:** Michel A. Struwe, Axel J. Scheidig, Bernd Clement

**Affiliations:** 1 Zoologisches Institut – Strukturbiologie, Christian-Albrechts-Universität Kiel, Kiel, Germany; 2 Pharmazeutisches Institut, Christian-Albrechts-Universität Kiel, Kiel, Germany

**Keywords:** molybdenum, metalloprotein, enzyme, liver, drug metabolism, mARC, nitric oxide

## Abstract

The mitochondrial amidoxime–reducing component (mARC) is one of five known molybdenum enzymes in eukaryotes. mARC belongs to the MOSC domain superfamily, a large group of so far poorly studied molybdoenzymes. mARC was initially discovered as the enzyme activating *N*-hydroxylated prodrugs of basic amidines but has since been shown to also reduce a variety of other *N*-oxygenated compounds, for example, toxic nucleobase analogs. Under certain circumstances, mARC might also be involved in reductive nitric oxide synthesis through reduction of nitrite. Recently, mARC enzymes have received a lot of attention due to their apparent involvement in lipid metabolism and, in particular, because many genome-wide association studies have shown a common variant of human mARC1 to have a protective effect against liver disease. The mechanism linking mARC enzymes with lipid metabolism remains unknown. Here, we give a comprehensive overview of what is currently known about mARC enzymes, their substrates, structure, and apparent involvement in human disease.

Early works of our group in the 1980s and 1990s had shown that basic amidines like benzamidine (BA) can be oxidized by microsomal P450 monooxygenases (1), but that the resulting amidoxime products, for example, benzamidoxime (BAO), can also be reduced back to their corresponding amidines by an unknown enzyme (2). *In vivo*, the reduction appears to be predominant (3). Amidines and amidoximes have very different physicochemical properties: while amidines are strongly basic and therefore form positively charged amidinium ions in an aqueous environment, the basicity of amidoximes is strongly decreased. This fact allows to administer amidoximes as prodrugs of amidines, which are much more readily absorbed in the intestine, but reduced to their corresponding amidines very quickly after absorption (4). This was demonstrated very early for the antiprotozoal drug pentamidine (3, 5). This prodrug principle was then used extensively when novel oral anticoagulants, which required an amidine moiety in order to bind to their targets’ active sites (6), were developed (7, 8, 9, 10, 11, 12). Ximelagatran, the first novel oral anticoagulant to enter the market used the amidoxime prodrug principle (13). However, at this point it was still not clear, which enzyme actually catalyzed the reduction of amidoximes to amidines. Figure 1*A* illustrates the oxidation of the model compound BA to BAO by P450 monooxygenases and the *retro*-reduction to BA by the mitochondrial amidoxime–reducing component (mARC) enzyme system.

**Figure 1:**
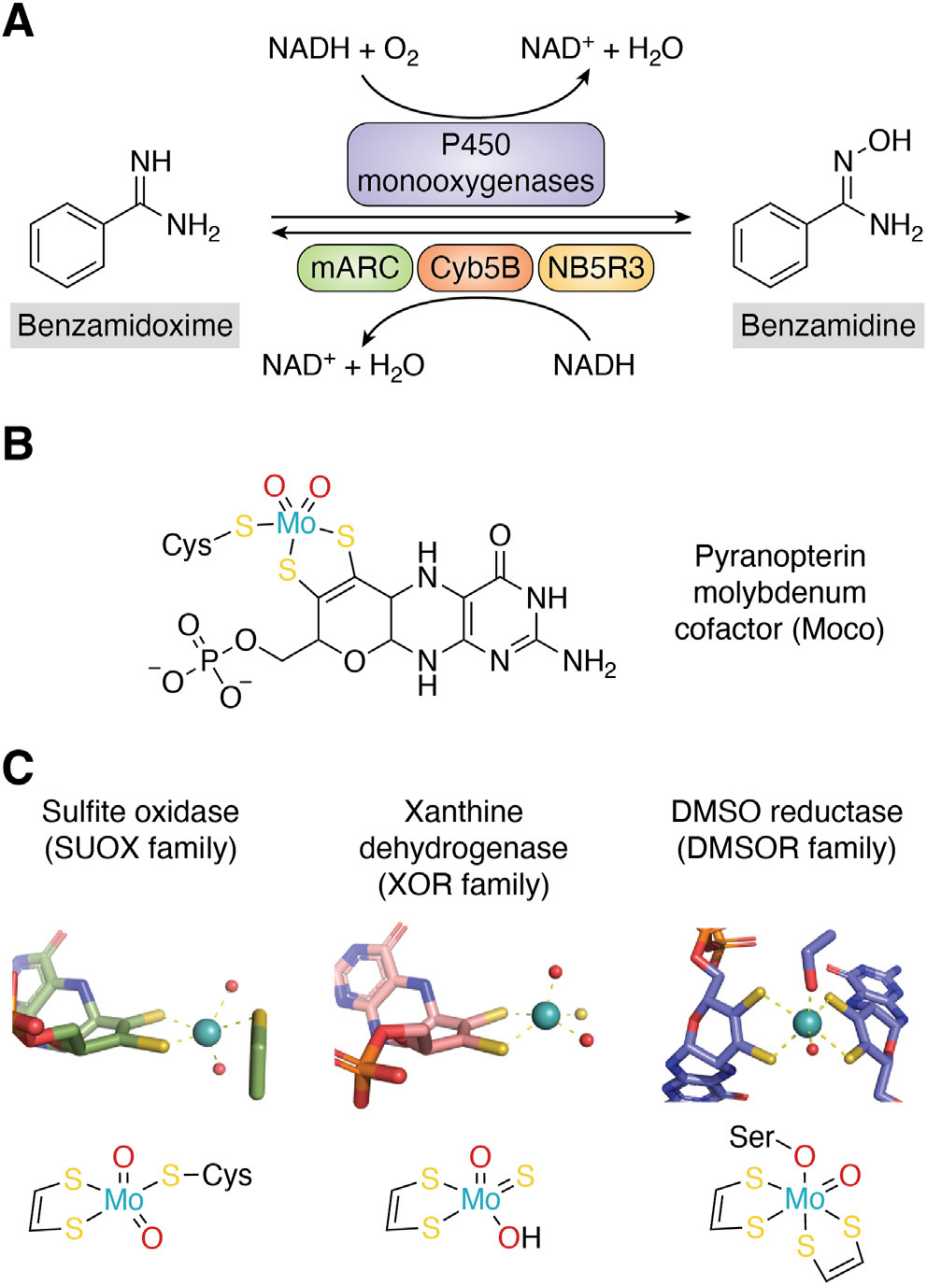
**Example of a reaction catalyzed by mARC and chemical structures of pyranopterin-molybdenum cofactors.***Panel A*, schematic representation of the oxidation of benzamidoxime to benzamidine by P450 monooxygenases and *retro*-reduction by the mARC enzyme system. *Panel B*, chemical structure of the pyranopterin molybdenum cofactor (Moco) utilized by human mARC enzymes. *Panel C*, molybdenum coordination at the active sites of stereotypical molybdenum enzymes: *Gallus gallus* sulfite oxidase (PDB: 1sox; SUOX family), *Bos taurus* xanthine oxidase (PDB: 3nvy; XOR family) and *Rhodabacter* capsulatus DMSO reductase (PDB: 1dmr; DMSOR family). mARC, mitochondrial amidoxime–reducing component; XOR, xanthine oxidoreductase.

An *N*-reducing enzyme system comprising cytochrome b5 (CYB5) and a flavin-containing NADH-CYB5 reductase (NB5R3) had been described much earlier, but the third component of this enzyme system remained elusive for some time (14). Reduction of *N*-hydroxylated compounds is oxygen-insensitive (15, 16, 17, 18), which is in excellent agreement with the mechanism of molybdenum (Mo)-containing oxotransferases proposed by Holm in 1990 (19). Finally, mARC was isolated from porcine liver mitochondria in 2006, identified by mass spectrometry (20) and found to be a novel Mo enzyme (21, 22). The history of mARC’s discovery has recently been described elsewhere in extensive detail (23).

mARC was the fifth eukaryotic Mo enzyme to be discovered after sulfite oxidase (SUOX), xanthine oxidoreductase (XOR), aldehyde oxidase (AOX), and plant nitrate reductase (20). All mammalian genomes encode two mARC paralogs, which are referred to as mARC1 and mARC2 (gene names *MTARC1, MTARC2* or *Mtarc1, Mtarc2* for mice genes). Human mARC1 and mARC2 share approx. 66% sequence identity with each other (22) and 25% sequence identity with the Mo-binding portion of human molybdenum cofactor sulfurase (MOS) (24). Other animals possess only a single mARC protein, for example, the zebrafish *Danio rerio* (25). Proteins equivalent to mARC have also been described in green algae and higher plants. For example, the green algae *Chlamydomonas reinhardtii* possesses a single protein, which is approx. 26% identical to human mARC proteins. Since it is not associated with mitochondria like the human mARC proteins, it is referred to as amidoxime reducing component (ARC) or *Chlamydomonas reinhardtii* amidoxime–reducing component (crARC) (26). Higher plants again have multiple proteins similar to mARC, which are also not associated with mitochondria. Examples are the ARC1 and ARC2 proteins of *Arabidopsis thaliana*, which are approx. 30% identical to human mARC and have been purified and characterized (27). It should be stressed that human mARC1 does not correspond to plant ARC1, but phylogenetic analyses show that mammals and plants have developed their multiple mARC/ARC paralogs independently from one another (28). It has been suggested that bacterial YcbX proteins (29) are orthologs of mARC and should be grouped in the same subfamily within the larger molybdenum cofactor sulfurase C-terminal (MOSC) domain superfamily (*vide infra*) (30).

While the experiments culminating in the discovery of mARC were initially motivated by its property to metabolize xenobiotics and pharmaceutical drugs in particular, recent studies showed that a mARC2 KO has dramatic effects on lipid metabolism in a murine KO model (31) and that protein variants of human mARC1 convey a protective effect against diseases of the liver (32).

Our perspective on mARC enzymes has therefore shifted quite significantly in recent years: interest in the enzyme was sparked initially by its property to activate amidoxime prodrugs. Afterward, many other *N*-reductive biotransformation reactions of drug substances and toxic compounds were studied. Now, the focus lies on the involvement of mammalian mARC enzymes in lipid metabolism and human disease. This review aims to give a comprehensive overview about our current understanding of mammalian mARC enzymes and their homologs from other species, their relationship with each another, and possible physiological functions.

## A brief overview of Mo enzymes

Mo is an essential element for life on earth. It is crucial for fixation of dinitrogen (N_2_) to ammonia (NH_3_) by nitrogenases, where the Mo is part of an iron-molybdenum cofactor (Moco) (33). All Mo enzymes other than nitrogenase are mononuclear and utilize a pyranopterin cofactor, whose dithiolene group coordinates a single Mo ion, thus forming the Moco (Fig. 1*B*). They typically catalyze two-electron oxidations or reductions by changing between the Mo(IV) and Mo(VI) oxidation states (34).

Mononuclear Mo enzymes are oftentimes classified into three main “families” based on the coordination environment of their catalytic Mo ion; these families are named after prototypical enzymes (34, 35). In members of the SUOX family, the catalytic Mo ion is coordinated by the dithiolene group of one Moco molecule, one conserved cysteine residue and two terminal oxygen ligands. Members of the XOR family do not have a cysteine ligand, but instead a terminal sulfido ligand. The Mo ion in dimethyl sulfoxide reductase (DMSOR) family members is usually hexacoordinated: dithiolene groups from two different Moco molecules bind to the Mo ion. Additionally, there is usually one amino acid ligand (cysteine, selenocysteine, aspartate, or serine) and one or multiple terminal oxo, sulfido, or selenido ligands. Figure 1*C* illustrates the active sites of typical Mo enzymes from each of these families.

Importantly, the distinct Mo coordination environments of the different Mo enzyme families can be used to catalyze redundant reactions. For example, *Escherichia coli* (*E. coli*) possesses two nitrate reductases, which belong to the DMSOR family. In the case of NarG, the Mo ion is coordinated by an aspartate sidechain (36), whereas in NapA a cysteine residue provides the sixth Mo ligand (37). DMSOR family members do not occur in eukaryotes. Plants and fungi still have molybdenum-containing assmilatory nitrate reductases, which instead belong to the SUOX family and have a completely different Mo coordination environment (38). Similarly, sulfoxides can be reduced both by members of the DMSOR family such as DmsA from *E. coli* (39) or MtsZ from *Haemophilus influenzae* (40, 41), but also by the MsrP/YedY proteins (42), which belong to the SUOX family.

Interestingly, many Mo enzymes are involved in human diseases. Deficiencies in Moco biosynthesis or SUOX lead to severe neurological disorders (43, 44), whereas XOR is critical in the pathogenesis of gout (45). Microbial Mo enzymes can be virulence factors for pathogenic bacteria (46).

## The MOSC domain superfamily

**Figure 2:**
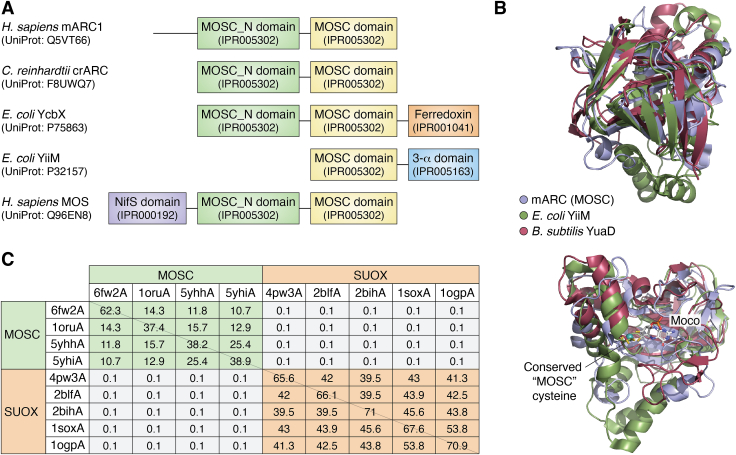
**Domain architecture and structural comparisons of MOSC domain proteins.***Panel A*, domain architectures of some representative MOSC domain proteins. UniProt IDs and *InterPro* IDs of the individual domains are given. *Panel B*, the mARC1 (PDB: 6FW2, *blue*) MOSC domain superimposed with *Escherichia coli* YiiM (PDB: 5YHI, *green*) and *Bacillus subtilis* YuaD (PDB: 1ORU). Proteins are shown in *cartoon representation*. The conserved “MOSC cysteine” residue and the Mo-molybdopterin cofactor of human mARC1 are shown in *stick representation*. *Panel C*, *DALI* structural superposition statistics of experimentally determined structures of MOSC domain proteins and SUOX family members. Table headers indicate the PDB accession IDs (6fw2A, *Homo sapiens* mARC1; 1oruA, *Bacillus subtilis* YuaD; 5yhhA, *Geobacillus stearothermophilus* YiiM; 5yhiA, *Escherichia coli* YiiM; 4pw3A, *Sinorhizobium meliloti* SorT; *Starkeya novella* SorB; 2bihA, *Pichia angusta* NR; 1soxA, *Gallus gallus* SUOX; 1ogpA, *Arabidopsis thaliana* SUOX). “*The Z-Score is a measure of quality of the alignment—the higher, the better. As a general rule, Z-scores above 8 yield very good structural superimpositions, Z-scores between 2 and 8 indicate topological similarities, and Z-scores below 2 are not significant.”* (200). Good structural superpositions of MOSC domain proteins and SUOX family members are found within their respective groups, but not between the two. mARC, mitochondrial amidoxime–reducing component; MOSC, molybdenum cofactor C-terminal; SUOX, sulfite oxidase.

In 2002, Anantharaman and Aravind described a large superfamily of proteins homologous to the C terminus of MOS. They accordingly named this region MOSC domain (47). Based on the known function of MOS (48), they assumed that proteins possessing a MOSC domain would constitute “metal-sulfur cluster biosynthesis proteins” (47). The *InterPro* database (49) currently lists >59,000 proteins containing a MOSC domain (IPR005302). MOSC domain proteins are extremely diverse with oftentimes hardly any detectable sequence identity between members of different subfamilies (30) and there is only a single ultimately conserved cysteine residue that all members of this family share (47). Also, they can have various different additional protein domains (*vide infra*, Fig. 2*A*).

Using the “canonical” definition of Mo coordination (*vide supra*), MOSC domain proteins should be classified as SUOX family members due to their Mo coordination environment, and this label is oftentimes applied to them. It should be noted though that MOSC domain proteins are not homologous to enzymes from the SUOX family in their amino acid sequences and there is no detectable structural similarity either (Fig. 2*C*). Thus, the similar Mo coordination is the only common attribute of MOSC domain proteins and SUOX family members, which indicates that classification of Mo enzymes solely based on the coordination of their catalytic Mo ion might be too superficial. It has instead been suggested to treat MOSC domain proteins as a separate family of Mo enzymes (30, 50).

Looking at MOSC domain proteins as an independent family of Mo enzymes, it has been found that they are the most common family of Mo enzymes in eukaryotes, with approx. 96% of Mo-utilizing eukaryotic organisms encoding at least one MOSC domain protein in their genomes. Similarly, MOSC domain–containing proteins are the second most common type of Mo enzyme in bacteria with approx. 79% of Mo-utilizing bacteria, possessing at least one MOSC domain protein. Prevalence of MOSC domain proteins in archaea is much more limited (51). Reportedly, MOSC domain proteins occur predominantly in microorganisms that favour aerobic conditions (51).

In their initial study, Anantharaman and Aravind identified several subfamilies of MOSC domain proteins with characteristic domain architectures: proteins of the YiiM subfamily occur mostly in prokaryotes but have also been identified in fungi (28) and are characterized by a small 3-α domain, which is fused to the C terminus of the MOSC domain and comprises a small bundle of three α-helices (47). The *InterPro* (49) database further contains YiiM-like proteins consisting of a MOSC domain, a 3-ɑ domain, and additional domains binding flavins and [2Fe-2S] clusters (example UniProt: Q89NR8). Additional groups of small MOSC domain proteins like the YuaD proteins that occur exclusively in prokaryotes with no domains other than the MOSC domain have furthermore been described (47). Lastly, Anantharaman and Aravind describe a “PA3022-like” family that we will refer to as YcbX-like proteins, an “FLJ22390-like” family, which we will refer to as mARC-like proteins and a MOS group (47). The latter three families have in common that the MOSC domain is fused to a β-strand–rich MOSC_N domain. YcbX proteins occur only in prokaryotes and typically possess a small ferredoxin domain carrying a [2Fe-2S] cluster but can also have an flavin-adenine dinucleotide (FAD)-binding domain fused between the MOSC and ferredoxin domains (52). mARC proteins may possess transmembrane helices and targeting sequences at their N terminus. MOS possesses a NifS domain at its N terminus, which is used to abstract sulfur from l-cysteine and transfer it to Moco bound at the MOSC domain (53).

It should be noted that some additional domain fusions of the MOSC domain can be found, for example, fusions with MoaB (UniProt: A6BK61) or XdhC (UniProt: C4ZDB5), which indicate a link to Moco biosynthesis. However, these proteins seem to be quite rare and have not been studied experimentally, yet.

## Catalytic properties of mARC

mARC was originally found through its property to catalyze two-electron reductions of *N*-oxygenated compounds like BAO (20). Its bacterial homologs YiiM and YcbX catalyze the same type of reaction (29, 54, 55). For all MOSC domain proteins that have so far been studied in *in vitro*, activity assays demonstrate this *N*-reductive activity. This even includes the C-terminal domain of MOS (20)—for which we know for sure that it has a different physiological function.

Thus, while Mo-dependent *N*-reduction definitively is a property of MOSC domain proteins and has been shown *in vitro* using recombinant enzymes as well as *in vivo* using KO models (31), it is uncertain whether this activity is at all related to their biological function. The physiological substrate of mARC, and other MOSC domain proteins for that matter, might be some entirely different compound, which has so far just not been considered.

The Mo(IV)/Mo(V) and Mo(V)/Mo(IV) midpoint potentials of human mARC proteins have been determined by electrochemical titrations as + 64 ± 10 mV and – 30 ± 10 mV for recombinant human mARC1 at pH 6. The potentials for human mARC2 are markedly lower at – 37 ± 10 mV and – 137 ± 10 mV, respectively (56). So, despite the high degree of similarity between human mARC1 and mARC2, the redox properties of their Mo active sites are quite different. Using the same method, the Mo(VI)/Mo(V) and Mo(V)/Mo(IV) midpoint potentials of *E. coli* YiiM were determined to be approx. – 30 mV and – 210 mV (54). Overall, these potentials seem to be in a similar range like those of other Mo-containing reductases like eukaryotic nitrate reductase (57, 58) or bacterial DMSO reductase (59).

The different redox potentials of human mARC1 and mARC2 might serve as an explanation for the slight differences in their catalytic properties. Importantly, human mARC1, but not human mARC2, reduces *N*-oxides (60, 61). *E. coli* YiiM also does not reduce *N*-oxides, and it appears to be more similar to mARC2 than to mARC1 in terms of redox potential (54). Nonetheless, not many MOSC domain proteins have been characterized electrochemically and a more comprehensive dataset would likely be required in order to identify clear links between redox potentials and substrate specificity.

## Substrates of mARC

The mARC enzyme system was shown to catalyze the reduction of a large variety of *N*-hydroxylated compounds such as amidoximes (21, 62, 63), *N*-hydroxy guanidines (64), *N*-hydroxy sulfonamides (65), *N*-hydroxylated nucleobase analogs (66), oximes (60), *N*-hydroxy amidinohydrazones (60), *N*-oxides (60, 61), hydroxylamines (67), and hydroxamic acids (68).

Several aspects are worth mentioning, most functional groups are reduced by both mARC paralogs with similar turnover rates. Important outliers are *N*-oxides (60, 61) and *N*-hydroxyurea (69), which have a strong preference for mARC1. Hydroxamic acids are also reduced by mARC1 with higher turnover rates than by mARC2 (68).

It has been tried to identify clear structure-activity relationships for mARC enzymes, for example, using a variety of *para*-substituted derivatives of the model substrate BAO (62), but so far, it remains difficult to predict whether a new compound might be reduced by mARC or not based on the chemical structure. Any compound containing an N–O bond should be considered a potential candidate for reduction by mARC enzymes. Recently established protocols for identification of mARC substrates by a photometric assay (69), an improved fluorescence-based protocol (70) or cyclic voltammetry (56, 71) will allow testing of potential substrates at much higher throughput than previously applied HPLC-based protocols.

## mARC as a drug-metabolizing enzyme

mARC research was initially motivated by *N*-hydroxylated prodrugs of compounds with strongly basic amidine and guanidine functional groups. *N*-hydroxylated prodrugs of various drugs incorporating these motives were synthesized. Increased bioavailability as well as rapid activation through-mARC catalyzed reduction were demonstrated. Examples include anticoagulants like ximelagatran (63) or dabigatran (72), the antiprotozoal agent pentamidine (73, 74), the antiviral drug oseltamivir (75), or the urokinase inhibitor upamostat (76) but also investigational drugs such as neuronal nitric oxide (NO) synthase (NOS) inhibitors (77). In many cases, reduction by mARC reverses oxidative biotransformation reactions catalyzed by P450 monooxygenases or flavin-containing monooxygenases (78).

In the case of prodrugs, reduction by mARC releases active metabolites. In other cases, *N*-hydroxylated compounds are the active drug substances and reduction by mARC leads to inactivation. Examples are hydroxamic acids like vorinostat or bufexamac, which require their hydroxamic acid moieties as “warheads” to bind to their metalloprotein targets and are reduced to inactive amides by mARC (68). Also, the cytostatic drug *N*-hydroxyurea is metabolized extensively by human mARC1 (56, 69), which might explain its very short biological half-life time and the very high doses required for this drug (79). We would like to point out that molnupiravir (Lagevrio), a prodrug of the mutagenic nucleoside analog ^4^*N*-hydroxycytidine (*vide infra*) is a mARC substrate (70) that has recently been approved for treatment of SARS-CoV2 (80). Plasma concentrations of the active metabolite ^4^*N*-hydroxycytidine (EIDD-1931) have a reported half-life time of only approx. One hour (81) and reduction by mARC1 potentially contributes to the rapid metabolism.

While many potentially toxic *N*-oxygenated compounds are reduced by mARC, some toxic metabolites are stable toward mARC-catalyzed *N*-reduction. This includes the hydroxamic acid metabolite of phenacetin (68), which is believed to be responsible for phenacetin’s toxic and mutagenic side effects (82). We suggest that specifically those *N*-hydroxylated compounds are toxic, which are not mARC substrates.

## Potential endogenous substrates

While initially a strong emphasis was placed on mARC enzymes as biotransformation enzymes catalyzing the reduction of xenobiotics, many potential endogenous substrates have been investigated, too. Trimethylamine *N*-oxide (TMAO) is correlated with increased risk of cardiovascular diseases. mARC1, but not mARC2, reduces TMAO, albeit with very poor turnover rates and very high K_m_ values (61). Given that other Mo enzymes, for example, TorA from *E. coli* can reduce TMAO with drastically higher turnover rates, we consider it unlikely that TMAO is the physiological substrate of mARC1. The potential involvement of mARC in NO biosynthesis through reduction of nitrite has been studied extensively (*vide infra*).

mARC’s bacterial counterparts YcbX and YiiM were initially discovered by screening transposon libraries for mutants sensitive toward the mutagenic nucleobase analogue 6-hydroxlaminopurine (6-HAP), which can be *N*-reduced to adenine (29). mARC enzymes, too, can protect eukaryotic cells against the mutagenic effects of *N*-hydroxylated nucleobase analogs like 6-HAP or ^4^*N*-hydroxycytosine and their equivalent nucleosides ^6^*N*-hydroxyadenosine and ^4^*N*-hydroxycytidine (66, 83). A similar function has been associated with *E. coli* YiiM and YcbX (29). Also, crARC activity in *C. reinhardtii* protects the algae against 6-HAP (26). Whether these compounds occur under physiological conditions, however, has been questioned (84). Recently, it was discovered that certain microbes, that is, *Staphylococcus epidermidis*, may synthesize 6-HAP (85). Human cells expressing mARC enzymes were found to be resistant toward 6-HAP, while it did have an antiproliferative effect on tumor cell lines not expressing mARC (85).

There are some reports correlating mARC with antioxidant effects (86). Therefore, mARC might also be involved in the synthesis or inactivation of reactive oxygen species or reactive nitrogen species. It has been described recently that human mARC enzymes are capable of reducing hydrogen peroxide (H_2_O_2_) to water (87). While there is no evidence that hydrogen peroxide itself is the physiological substrate of mARC, this proves that mARC’s substrate spectrum might go far beyond the *N*-oxygenated compounds that have been studied so far.

All in all, it is very difficult to state, which compound or compounds might be the physiological substrate of mARC enzymes. It is certainly true that many *N*-hydroxylated substances either xenobiotics or products of oxidative biotransformation can be reduced by mARC enzymes. However, we do believe that at this point available data is insufficient to point out specific substances as physiological substrates.

## Involvement in NO biosynthesis

All eukaryotic Mo enzymes, including SUOX, nitrite reductase, XOR, and nitrate reductase can under certain conditions reduce nitrite (NO_2_^−^) to NO (88). NO is a very important biological signaling molecule, involved in a variety of processes (89). In animals, NO can be generated *via* an oxidative pathway by NOS from l-arginine *via* a two-step mechanism: First, l-arginine is oxidized using molecular oxygen and forms the NO precursor *N*^ω^-hydroxy-l-arginine, which then reacts to l-citrulline and NO (90). Plants on the other hand seem to generate NO *via* a reductive pathway, specifically by one-electron reduction of NO_2_^−^ to NO using nitrate reductase (91), an enzyme that also catalyses the two-electron reduction of nitrate to nitrite. It has been suggested to consider mammalian Mo enzymes “nondedicated” NO synthases, which maintain NO levels under hypoxic conditions (92).

In 2014, Sparacino-Watkins *et al.* demonstrated that human mARC1 and mARC2 can reduce NO_2_^−^ to NO, both with recombinant proteins and in cell culture–based models. Nitrite reductase activity is highest at pH 6.5 and is inhibited significantly by oxygen (93, 94). Interestingly, for other Mo enzymes like XOR, AOX, and SUOX, similar pH optima have been described (95, 96). The acidic pH optimum of nitrite reduction is worth mentioning, since, for example, in case of SUOX, the optimum for the canonical function, oxidation of sulfite to sulfate, is at much higher pH (97). It has thus been suggested that the sulfite oxidizing and nitrite-reducing activity of SO are regulated in a pH-dependent manner (98).

Using electron paramagnetic resonance (EPR) spectroscopy, Yang *et al.* (99) demonstrated that fully reduced ARC2 from *A. thaliana* (Mo(IV)) is quantitatively oxidized to a paramagnetic Mo(V) species upon incubation with excess nitrite under anaerobic conditions, confirming a one-electron reduction. Similar observations were also made with SUOX (95).

The crARC protein from *C. reinhardtii* is involved in NO—-dependent NO synthesis and expression of crARC in the algae is induced by nitrite. The enzyme appears to utilize different electron transport partners for reduction of nitrite and *N*-hydroxylated substrates (100) (*vide infra*). On the other hand, a recently published study on the NOS activity of *A. thaliana* ARC1 and ARC2 only found very low NO—-reducing activities for these enzymes. Furthermore, knockout of the *ARC1* and *ARC2* genes does not seem to impact tissue NO concentrations *in vivo*. The authors conclude that in *A. thaliana*, nitrite reductase and not ARC proteins is responsible for NO synthesis from nitrite (27).

While *in vitro* experiments indicate that mARC enzymes, just as all other eukaryotic Mo enzymes, can reduce NO_2_^−^ to NO, it is not clear whether or not this reaction is in any way related to the physiological function of mARC in human. In human ovarian cancer cells, supplementation of Na_2_MoO_4_ to the culture medium has been reported to increase intracellular NO concentrations and deplete GSH. This effect is decreased by knockdown of the *SUOX*, *AOX*, and *MTARC1* genes, while knockdowns of *XDH* and *MTARC2* have no influence (101). On the other hand, a preprint describing the synthesis of NO from NO_2_^−^ by human astrocyte mitochondria under anoxic conditions indicates that SUOX, not mARC, is responsible (102). Also, there is some evidence that expression of mARC1 and mARC2 might be downregulated under hypoxic conditions (103), under which NO_2_^−^ reduction would likely occur.

**Figure 3:**
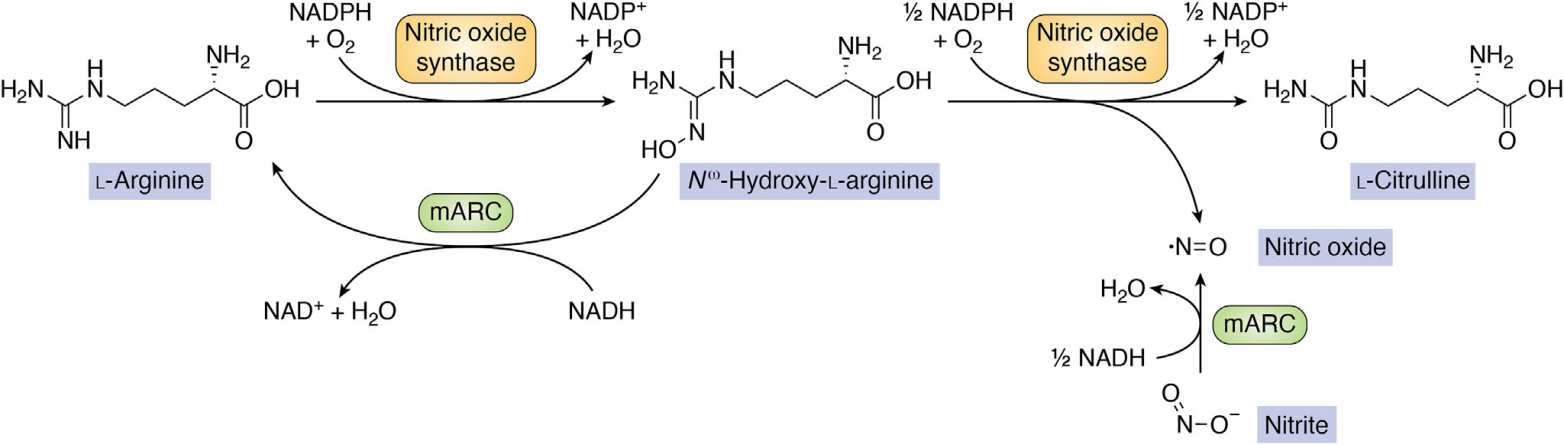
**Illustration of “canonical” nitric oxide biosynthesis by nitric oxide synthase and possible influences of mARC on nitric oxide homeostasis.** mARC, mitochondrial amidoxime–reducing component.

In conclusion, available data does not support NO—-dependent NO synthesis to be a physiological function of human mARC enzymes, while it might be relevant in other organisms, that is, algae. A different function of mARC with relation to NO is also conceivable: both mARC1 and mARC2 are capable of reducing the NO precursor *N*^ω^-hydroxy-l-arginine, which contains an *N*-hydroxylated guanidine moiety, to arginine (64) with high turnover rates and thus reverse the first step of oxidative NO biosynthesis. This could lead to decreased cellular NO levels. Figure 3 illustrates how mARC could influence NO homeostasis.

## Biophysical and spectroscopic characteristics of mARC

Mammalian mARC proteins have an N-terminal outer mitochondrial membrane (OMM)–targeting sequence as well as a transmembrane helix. When these regions are truncated, active, Mo-loaded proteins can be expressed recombinantly in *E. coli* (21, 22). The truncated recombinant mARC1 and mARC2 proteins are monomeric (22), the same has been described for crARC (104) and the *A. thaliana* ARC1 and ARC2 proteins (27). On the other hand, bacterial MOSC domain proteins may form homodimers. This has been demonstrated for *E. coli* YiiM using size exclusion (105) and can be deduced from the crystal structure of *Bacillus subtilis* YuaD using structure analysis tools like *EPPIC* (106). mARC proteins represent the simplest eukaryotic Mo enzyme, as they bind only Moco as a cofactor and no additional prosthetic groups (107).

Recombinant human mARC1 and mARC2 proteins possess a weak optical absorption feature at 350 to 400 nm and another shoulder at approx. 465 nm in their oxidized Mo(VI) form (22). The spectrum changes when the proteins are reduced to the Mo(IV) state (56) with the 465 nm in particular being observed only for the oxidized form (22). Very similar optical properties have been described for other MOSC domain proteins like the mARC homolog crARC from *C. reinhardtii* (26), *E. coli* YiiM (54, 105), and the C-terminal domain of MOS (108).

The excitation energies of these absorption features roughly correspond to those described for SUOX family members (38, 109, 110, 111, 112). It is noteworthy, that the absorption bands of the latter are usually much better resolved with clear absorption bands instead of the rather vague shoulders seen in the spectra of MOSC domain proteins. Bleaching of the absorption band of SUOX, leading to a spectrum more similar to that of human mARC, has been associated with mutations of residues interacting with the Mo site of human SUOX (97). The 350 to 400 nm absorption band is commonly assigned to the dithiolene → Mo charge transfer, while the band at 465 nm would be assigned to the Cys → Mo charge transfer in SUOX-type enzymes (113, 114).

When human mARC1 and mARC2 are partially reduced to the paramagnetic Mo(V) state, rhombic EPR signals with strong ^1^H hyperfine coupling can be observed, which strongly resemble the low-pH form of SUOX, (22, 56). This, like the similar features in optical absorption spectra, points toward a likely very similar Mo coordination. The EPR spectrum generated by incubation of reduced *A. thaliana* ARC2 with excess sodium nitrite again appears to be very similar (99). A detailed pulsed EPR study including ^2^*H* and ^17^*O* labeling with recombinant human mARC2 concluded that the Mo(V) EPR spectrum likely arises from a pentacoordinated Mo species with two equatorial dithiolene sulfur atoms, one axial oxo group, one equatorial hydroxo ligand, and one additional equatorial sulfur or oxygen-derived ligand (115). The EPR spectrum of *E. coli* YiiM on the other hand has been described as almost axial with no observable ^1^*H* hyperfine coupling, which might be explained by a slightly different geometry of the Mo complex (54).

Treatment of human mARC proteins with cyanide does not lead to release of thiocyanate or loss of catalytic activity (22), which would be typical for members of the XOR family that possess a terminal sulfur ligand (116). Additionally, all MOSC domain proteins share one ultimately conserved cysteine residue, which corresponds to Cys273 in human mARC1 and Cys272 in human mARC2. Substitution of this cysteine in *C. reinhardtii* crARC leads to complete loss of *N*-reductive activity. Curiously, initial site-directed mutagenesis experiments with human mARC proteins revealed no changes in the Mo(V) EPR spectrum when the conserved cysteine was mutated to a serine (22). It was only shown later that this finding was due to a contaminated bacterial culture and that the “true” p.C273S variant of human mARC1 shows a strongly perturbed EPR spectrum (24).

Consequently, it appears likely that in human mARC proteins, crARC, and perhaps all MOSC domain proteins, the catalytic Mo ion is pentacoordinated and strongly resembles that of SUOX, with a conserved cysteine residue as a Mo ligand.

**Figure 4:**
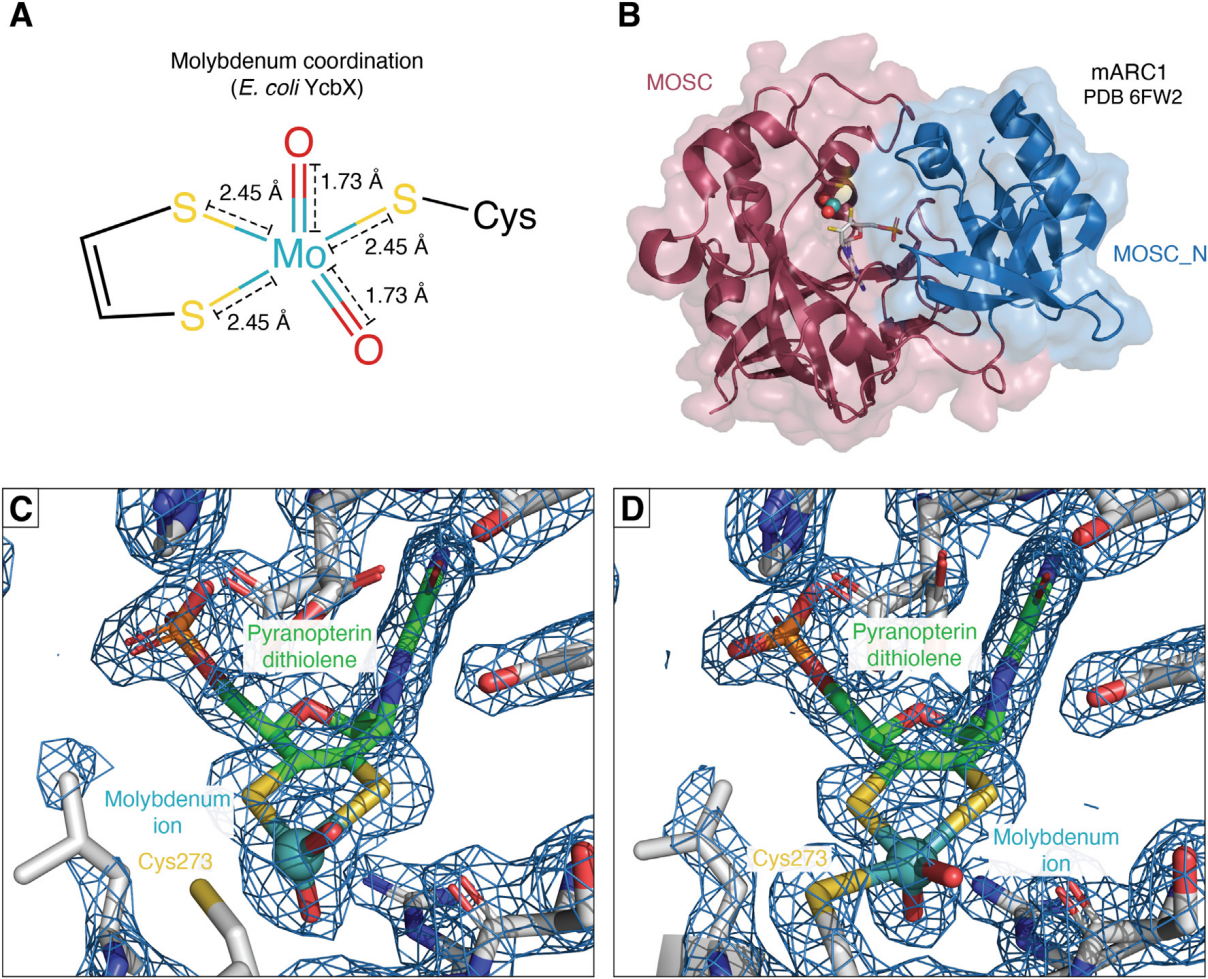
**Structure of the mARC molybdenum site.***Panel A*, Mo coordination in fully oxidized *Escherichia coli* YcbX according to extended X-ray absorption fine structure analyses (118). This agrees well with *Arabidopsis thaliana* ARC2 (117) and *H. sapiens* mARC1 (unpublished data). *Panel B*, protein structure of human mARC1 (PDB 6FW2). The protein is shown in *cartoon representation* with residues assigned to the MOSC_N domain (IPR005303) in *blue* and those assigned to the MOSC domain (IPR005302) in *red*. The Mo-MPT cofactor is shown in *stick representation*. The molecular surface in shown in transparent representation. *Panel C* and *D*, electron density maps at the active site of human mARC1 in the structure of (*C*) WT mARC1 (PDB: 6FW2, *thin needles*, microfocused X-ray beam) and (*D*) p.A165T variant protein (PDB: 7P41, *brick-shaped crystal*, collimated X-ray beam). 2F_O_-F_C_ maps are shown as a *blue mesh*, the contour level is 1σ. mARC, mitochondrial amidoxime–reducing component; MO, molybdenum.

Furthermore, *A. thaliana* ARC1 and the *E. coli* mARC homolog YcbX have been examined by X-ray absorption spectroscopy (117, 118). In both cases spectra again strongly resembles SUOX and the active site could be modeled with two sulfur ligands from the Moco’s dithiolene, one cysteine side chain, and two terminal oxygen ligands (Fig. 4*A*). The coordination distances of these terminal oxygen ligands identify them as oxo ligands. In reduced YcbX, there is one oxo ligand and one oxygen ligand with a longer coordination distance, possibly a hydroxo or aqua ligand. Since, as far as we know, mARC and YcbX prefer *N*-oxygenated substrates with an *-OH* group, a reaction mechanism based on a hydroxo active site involving a protonation step was proposed (118). In SUOX, high-resolution X-ray absorption analyses show that the equatorial oxygen ligand is a coordinated water in the Mo(IV) state (119). Further spectroscopic studies are required, but the nature of this ligand might constitute a difference between SUOX family members and MOSC domain proteins and explain their different reactivities.

## Protein structure of mARC

In the late 1990s and early 2000s, thousands of experimental structures of mostly prokaryotic proteins were made available through structural genomics initiatives (120). This included X-ray crystal structures the *E. coli* YiiM protein (121) as well as the *B. subtilis* YuaD protein, which belong to the MOSC domain superfamily and are distant homologs of eukaryotic mARC. Additionally, the structure of the *Bordetella bronchiseptica* protein BoR11 was determined by NMR spectroscopy (122). BoR11 is a distant homolog of mARC’s N-terminal domain.

Although Kozmin *et al.* were able to demonstrate that YiiM catalyzes *N*-reduction using Moco (29, 123), neither of the aforementioned structures showed any indications of cocrystallized cofactor. It should be noted however that expression of molybdoenzymes in their *holo*-form can be quite challenging (124). In most structural genomics projects investigating prokaryotic proteins, the popular *E. coli* strain BL21 is used as an expression host (121); this strain only produces small amounts of Moco (125). Indeed, when YiiM proteins from *E. coli* and *Geobacillus stearothermophilus* were studied more closely, only marginal amounts of Mo were copurified with protein batches expressed in the BL21 strain (105). Switching to the Moco accumulating TP1000 strain (126) lead to strongly increased Mo loading. However, Mo saturations remained <20% and the resulting structures contain no electron density for bound Moco (105).

It was attempted to predict the structure of *C. reinhardtii* crARC based on Bor11 and YuaD using homology modeling through *SwissModel* (127). In this predicted structure, the C-terminal MOSC domain binds the Moco, while the N-terminal MOSC_N domain is not close to the active site (104). Despite our best efforts, eukaryotic mARC proteins proved very stubborn to crystallize. We were unable to find any conditions suitable for crystallization of recombinant human mARC1 or mARC2. Eventually, diffraction quality crystals of human mARC1 were obtained using a fusion-protein approach, where T4 lysozyme was inserted between two predicted *β*-sheets (128). Phasing was achieved using the structure of T4 lysozyme and multiple partial homology models of mARC1 (129).

Human mARC1 constitutes the first and, so far, only MOSC domain protein, for which an experimental structure of the *holo*-enzyme with cocrystallized Moco is available. mARC1 appears to be compact and roughly globular with the MOSC and MOSC_N domains packed very tightly together (Fig. 4*B*). The Moco is bound in a tight crevice between the two domains and is stabilized by many hydrogen bonds and salt bridges (129). Despite the very low degree of sequence identity between mARC1, YiiM and YuaD, the structures of the proteins appear remarkably similar when they are superposed using *DALI* (Fig. 2, *B* and *C*) (130).

**Figure 5:**
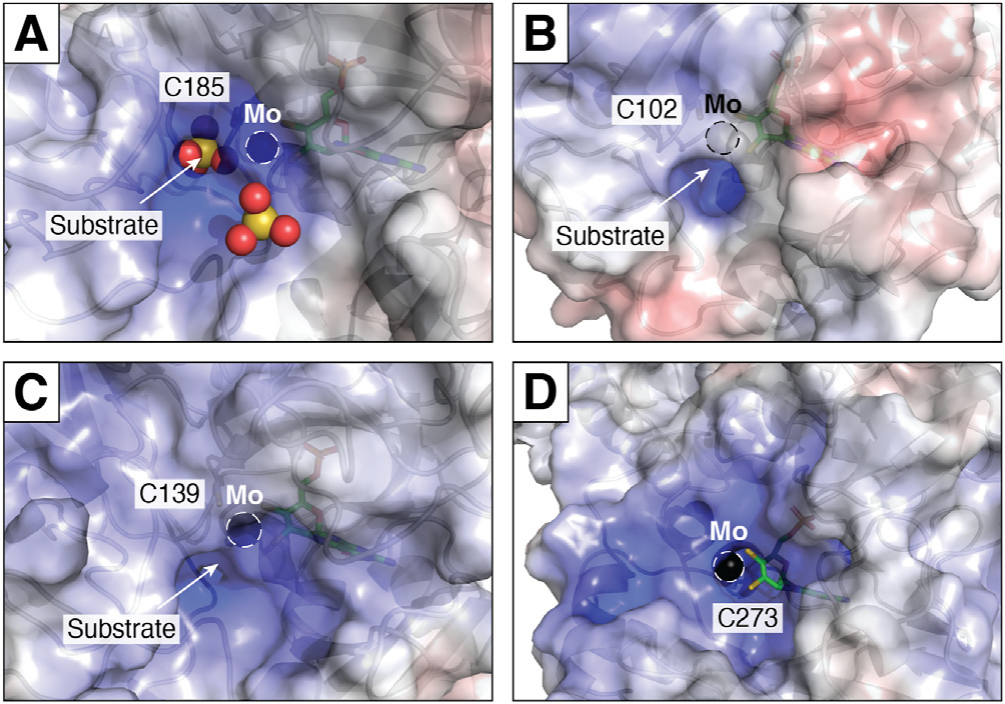
**Comparison of the substrate recognition sites of different molybdoenzymes.** Protein surfaces are colored according to their electrostatic surface potential (from *red*, 10 k_B_T/e_c_ over white 0 k_B_T/e_c_ to *blue* +10 k_B_T/e_c_). *Black spheres* represent the catalytic molybdenum ion. *Panel A*: chicken liver sulfite oxidase with two cocrystallized sulfate ions (PDB: 1SOX). *Panel B*: *Escherichia coli* MsrP (PDB: 1XDQ). *Panel C*: *Pichia angusta* nitrate reductase (PDB: 2BIH). *Panel D*: human mARC1. When compared to the other structures, the catalytic molybdenum ion of mARC1 is highly solvent-exposed, as is the coordinating residue C273. mARC, mitochondrial amidoxime–reducing component.

An important difference between the active site of mARC1 and Mo enzymes from the SUOX, XOR, and DMSOR families is the high solvent exposure of the catalytic Mo ion. In other Mo enzymes, the Mo ion is buried within a narrow, well-defined substrate binding pocket, whereas mARC1 does not appear to have a clear substrate-binding site, instead, the Mo ion is located in a large, bowl-like suppression and is highly solvent-accessible. Figure 5 depicts the active sites of mARC1 in comparison with SUOX family members, that is, *Gallus gallus* SUOX (131), *Pichia angusta* nitrate reductase (132), and *E. coli* YedY/MsrP (133). The high solvent exposure of the catalytic Mo ion might explain why mARC enzymes are very “promiscuous” with respect to their substrate spectrum and do not show clear structure-activity relationships.

When the electron density surrounding the Mo ion itself is inspected, another curious feature can be observed: spectroscopic studies had previously shown that the active site of mARC proteins is likely very similar to SUOX family members, with the Mo ion coordinated by two-terminal oxygen ligands, the Moco dithiolene group, and the conserved “MOSC cysteine,” which in case of mARC1 is Cys273 (*vide supra*). However, hardly any electron density supporting coordination of Cys273 to the Mo ion is observed in the crystal structure. On the other hand, there is no clear electron density supporting a different conformation of the Cys273 sidechain either. The dataset for the original mARC1 crystal structure was collected from very thin needles using a very intense double focussed X-ray beam (128). The high X-ray dose may have induced to serious radiation damage to the metal site, leading to desulfuration of the cysteine sidechain. Indeed, when the structure of the disease-relevant mARC1 p.A165T variant was determined later, the crystallization conditions were optimized to obtain much larger crystals and data collection using a common collimated X-ray beam was possible (134). The resulting structure shows Cys273 fully coordinated to the Mo ion (Fig. 4, *C* and *D*), supporting the radiation damage hypothesis. The reason why cysteine desulfuration appears to be an issue in crystallography of mARC1 but not SUOX family proteins might, again, be the much higher degree of solvent exposure of this residue in mARC1. When the solvent-exposed surface area of the Mo-coordinating cysteine is estimated for the different structures using *DSSP* (135), values between 7 and 11 Å^2^ are calculated for SUOX family proteins, as opposed to approx. 45 Å^2^ for human mARC1.

It has been established that not only the Mo ion itself but also the pyranopterin cofactor is crucial for the catalytic properties of Mo enzymes and that the pyranopterin itself can assume various different oxidation states (136). A large-scale analysis of the pyranopterin geometries observed in crystal structures of Mo enzymes revealed that the pyranopterin can adopt two different geometries, which are likely indicative of different oxidation states and that these geometries are typical for the different “canonical” Mo enzyme families (137). Interestingly, the crystal structure of human mARC1 displays a pyranopterin geometry, which is typical for XOR family members, despite the Mo coordination resembling SUOX family proteins (129). This, again, supports our opinion that MOSC domain proteins such as mARC should not be treated as SUOX family members, but rather as a distinct family of Mo enzymes.

## mARC enzymes are not standalone proteins

Long before the mammalian mARC enzymes themselves were discovered, it was already known that the *N*-reducing enzyme system comprises CYB5 and NB5R (14, 15, 138). Mammalian cells encode multiple isoforms of these electron carrier proteins. For CYB5, there is a microsomal isoform (CYB5A) and a mitochondrial isoform (CYB5B). Cell culture studies using siRNA mediated knockdown of either isoform clearly showed that the CYB5 isoform required for mARC activity is the mitochondrial isoform CYB5B using both human (139) and murine (140) cell lines.

The situation was initially less clear concerning the relevant NB5R isoform. Mammalian genomes encode three isoforms of NB5R, referred to as NB5R1, NB5R2, and NB5R3. First siRNA knockdown experiments with murine cell cultures showed no significant reduction in *N*-reductive activity resulting from knockdown of either isoform, despite mRNA levels being decreased by approx. 80% (140). However, only very low amounts of NB5R3 are required for *N*-reductive activity. The optimal stoichiometry between CYB5 and NB5R3 was determined to be 10:1. But even at a 100:1 ratio, activities are still comparable when recombinant proteins are used. It was demonstrated in a human cell culture model using a more efficient *NB5R3* knockdown that NB5R3 is the main reductase in the *N*-reducing enzyme system (141).

Chamizo-Ampudia *et al.* were able to produce recombinant crARC as well as multiple potential electron carrier proteins, including five different CYB5 homologs and two potential flavin-containing reductases. It was shown by *in vitro* activity assays that crARC requires the hemoproteins crCyt b5-1 and the flavoprotein crCyt b5-R as electron carrier proteins for *N*-reductive activity and that this activity is increased dramatically by addition of Zn^2+^ ions (26). The authors further remarked that recombinant human mARC proteins are much less active than native proteins isolated from porcine liver samples and suggest that perhaps Zn^2+^ ions are also required by the mammalian *N*-reducing enzyme system proteins for optimal activity. We have not been able to confirm this hypothesis with our own recombinant human enzymes (unpublished data).

Indeed, interactions between crARC and its electron carrier proteins are stable enough for higher molecular weight complexes to elute in size-exclusion chromatography experiments after coincubation of the individual proteins (104). The binary complex of crARC with crCyt b5-1 appears to consist of one molecule crARC and two molecules crCyt b5-1, while crARC and crCyt b5-R appear to form a 1:1 complex. When all three proteins are analyzed together, a large complex of >100 kDa was observed but the exact stoichiometry of this complex could not be determined unambiguously (104). The soluble plant homologs of mARC from *A. thaliana*, ARC1 and ARC2, can act as reductases of *N*-hydroxylated compounds in concert with *A. thaliana* CYB5 isoform A and CYB5 reductase isoform 1 again displaying an electron transport chain very similar to that of human mARC proteins (27). Thus, electron transport from a NB5R-like flavoprotein to a CYB5B and then to mARC/ARC seems to be a universal feature of eukaryotic mARC/ARC enzymes.

The situation is a little different with *E. coli* YcbX, a bacterial enzyme, which has approx. 48% sequence similarity with human mARC1 (30). In this case, a spinach-type ferredoxin domain binding a [2Fe-2S] cluster is fused to the C terminus of the protein (29). This ferredoxin domain likely fulfils the function of the CYB5 in the eukaryotic mARC/ARC complexes. YcbX additionally requires the protein CysJ (52), a flavoprotein, elsewise involved in formation of the sulfite reductase complex together with the haemoprotein CysI (142). There are other bacterial YcbX proteins, that is, from *Vibrionales*, which comprise a full electron transport chain consisting of an Mo-binding domain, an NAD(P)H/FAD-binding domain and a [2Fe-2S]-binding ferredoxin domain into a single-protein chain (52).

Curiously, there is an alternative electron transport chain for crARC, which is preferred specifically for the reduction of nitrite to NO (*vide supra*), where the FAD-binding and haem-binding domains of the molybdoenzyme nitrate reductase are the electron transport partners of crARC (100). The findings regarding crARC and its interaction with nitrate reductase are extremely interesting, as they demonstrate that mARC-like MOSC domain proteins might utilize multiple different partners for electron transport and that substrate specificity may depend on the electron transport partners. In its function as a nitrite-dependent NOS, crARC is referred as NO-forming nitrite reductase. Due to its variable activity with different electron transport partners, crARC is regarded a “moonlighting” protein (28, 143).

## Tissue distribution of mARC in mammals

According to the human protein atlas’ (144) tissue consensus dataset, *MTARC1* mRNA levels are highest in adipose tissue, followed by the breast, liver, and thyroid. *MTARC2* on the other hand shows highest transcript levels in the liver, followed by kidney and parathyroid gland tissues. This is in agreement with data reported elsewhere, according to which both mARC1 and mARC2 are expressed in adult human liver. The same authors also reported that *MTARC1* expression levels are similar in liver and adipose tissue, whereas comparably only very little *MTARC2* was detected in adipose tissue (145). A recent targeted proteomics-based study showed that both mARC1 and mARC2 proteins are abundant in the liver, but mARC2 concentrations in kidney tissue were significantly higher (146).

Immunoblot studies on different murine tissues found the high levels of mARC1 expression in the liver, kidney, and pancreas. mARC2 on the other hand was expressed in the liver and kidney at approx. the same level, but also showed substantial expression in thyroid, lung, small intestine, and pancreas; adipose tissue was not examined in this study (66). Other authors reported, that mARC2 rather than mARC1 is the dominant mARC paralogue in rat adipocytes (140).

Thus, the tissue-specific expression profile of mARC enzymes probably differs between species. Consequently, it is very difficult to estimate, to which degree mARC1 and mARC2 are isofunctional. While all mammalian genomes encode for two mARC paralogs, other animals, like the zebrafish *D. rerio* only possess a single mARC enzyme to begin with (25). BLAST (147) searches of the UniProt (148) database indicate the same to be true for many birds, reptiles, and amphibia. Therefore, mammalian mARC enzymes might well be performing the same role, perhaps in different tissues and under different regulation. Alternatively, mARC paralogs might have acquired additional functions in mammals during evolution.

## Subcellular localization

Mammalian mARC proteins were first isolated from porcine OMM preparations (20). Studies in human cell culture with GFP-tagged mARC constructs confirmed localization in the OMM with the N terminus oriented toward the intermembrane space and the C terminus toward the cytosol, which is in agreement with the localization and orientation of their electron transport partners CYB5B and NB5R3 (149).

Some studies indicate that mARC proteins might also be localized in peroxisomes (150, 151). It has been speculated that an arginine-rich sequence located downstream of the transmembrane helix might represent a peroxisomal-targeting sequence (24). To our knowledge, CYB5B and NB5R3 are not present in peroxisomes. Peroxisomal localisation of mARC proteins and the function that mARC might perform there remain poorly understood.

## mARC enzymes in lipid metabolism and liver disease

A potential connection between energy homeostasis, particularly lipid metabolism and mARC enzymes has been known for some time now. For example, an intron variant of the *MTARC1* was among 95 other loci identified by a 2010 genome-wide association study (GWAS) searching for variants associated with serum lipids (152). Furthermore, significant *N*-reductive activity was detected in rat adipose tissue (138), even before the mARC protein itself had been isolated. When Neve *et al.* studied differentiation of rat 3T3-L1 fibroblasts to adipocytes, they observed a strong upregulation of the *MTARC2* and *NB5R* genes. *si*RNA-mediated downregulation of *MTARC2* lead to decreased lipid content of the adipocytes (140).

An *in vivo* study then revealed that in mice, liver concentrations of mARC1 and mARC2 proteins as well as *N*-reductive activities were decreased under fasting conditions but increased upon exposure of the animals to high-fat diet. Curiously, the *MTARC1* and *MTARC2* mRNA levels changed in the opposite direction, implying posttranslational regulation of intracellular mARC protein concentrations (153). Other studies have shown decreases in both hepatic *MTARC1* and *MTARC2* mRNA concentrations in mice after 24 weeks on a TD190883 (obesity model) diet (154). Regulation of mARC in response to nutritional state could also be demonstrated in humans: mARC2 protein concentrations in adipose tissue of obese patients decrease in response to a calory-restricted diet (145). Further, it was shown in a small study that mARC1 protein levels in adipose tissue are changed in patients with gestational diabetes mellitus compared to healthy controls (155). It has also been proposed that regulation of the *MTARC2* gene in kidney is related to type-2 diabetes (156).

A very significant impact of mARC2 on murine lipid metabolism became obvious, when *MTARC2* KO mice were characterized. The *MTARC* KO made the animals resistant toward body weight increase induced by high-fat diet. Furthermore, the mice had decreased serum cholesterol levels (31). Another group, which studied the same mouse strain (C57BL/6NmARC2^tm24^), confirmed the effect on body weight under high-fat diet. They also demonstrate lower body fat. At the same time, the *MTARC2* KO mice consume more food but are more active and have increased energy expenditure (157), which is in agreement with a reported increased body temperature (31).

The apparent involvement of mARC enzymes in lipid metabolism has received very much attention after a GWAS reported a common polymorphism of the *MTARC1* gene, specifically, the mARC1 p.A165T variant, to be correlated with non-alcoholic fatty liver disease (NAFLD) and liver cirrhosis (32, 158). Multiple similar studies have since confirmed, that carriers of this variant have a decreased risk of liver cirrhosis (158, 159, 160, 161, 162), lower levels of liver fat (32, 163, 164, 165, 166) and a characteristically changed profile of serum lipids and circulating markers of liver injury (see Table 1). Taken together, there is conclusive evidence that the mARC1 p.A165T variant conveys protection against NAFLD and its progression toward nonalcoholic steatohepatitis (NASH). In fact, carriers of this variant show reduced liver-related mortality, especially when additional risk factors (high body mass index, type-2 diabetes) are involved (166). A Korean study found no effect of the mARC1 p.165T variant on the risk of liver disease in lean individuals, confirming that mARC1 is specifically important when additional risk factors, that is, obesity are involved (167). The mARC variant might even influence the risk of hepatocellular carcinoma (HCC) (161, 162).

**Table 1:** Overview of surrogate parameters relevant for metabolic syndrome and liver disease, which are changed in carriers of the protective p.A165T variant compared to carriers of the WT allele in different genome-wide association studies

Citation	ALT	AST	ALP	TC	HDL	LDL	TG
Emdin *et al.* 2020 (28)	↓	↓	↓	↓	↓	↓	↑
Emdin *et al.* 2021 (151)	↓	/	/	/	/	/	/
Gao *et al.* 2021 (191)	↓	↓	/	/	/	/	/
Janik *et al.* 2021 (111)	↓	↓	↓	/	/	/	/
Schneider *et al.* 2021 (157)	↓	↓	↓	↓	↓	↓	↑
Fairfield *et al.* 2022 (155)	↓	↓	↓	↓	↓	↓	↑
Rivera-Paredez *et al.* 2022 (192)	/	/	/	/	/	/	↑
Sveinbjornsson *et al.* 2022 (153)	↓	↓	↓	↓	↓	↓	↑

↓ = lower levels; ↑ = increased levels; / = not reported in this study.

ALP, plasma alkaline phosphatase activity; ALT, plasma alanine transaminase activity; AST, plasma aspartate aminotransferase activity; HDL, plasma HDL cholesterol; LDL, plasma LDL-cholesterol; TC, total plasma cholesterol; TG,plasma triglycerides.

NAFLD is an extremely common disease, with a prevalence of >30% worldwide (168). NAFLD is strongly associated with the metabolic syndrome and can progress to NASH, liver cirrhosis, and eventually HCC (169). Due to its ever-rising prevalence, NAFLD-NASH has been referred to as an “under-recognized epidemic” (170). Even though it is estimated that only approx. 10% of patients diagnosed with NAFLD develop cirrhosis or HCC within 20 years after diagnosis, the high prevalence of NAFLD still results in a significant disease burden (171). There is currently no effective therapy against NAFLD, therapeutic strategies thus mostly rely on lifestyle interventions (170).

**Figure 6:**
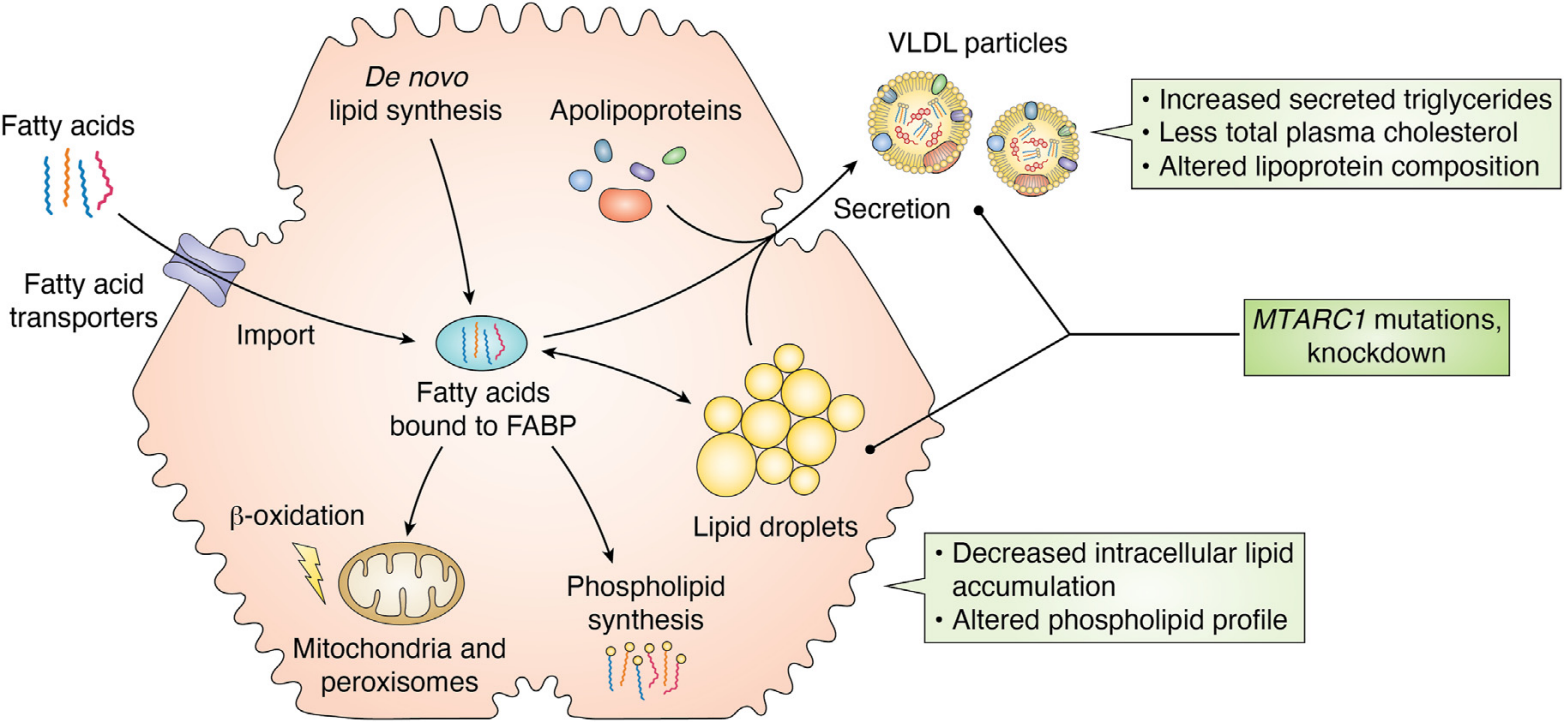
**Highly simplified representation of triglyceride metabolism in hepatocytes and changes induced by mARC protein variants in humans or *MTARC1* knockdown in primary hepatocytes.** mARC, mitochondrial amidoxime–reducing component.

The most important feature of NAFLD is accumulation of triglycerides as lipid droplets within the hepatocyte cytosol (172). Triglycerides are formed by esterification of glycerol with fatty acids (FA). There are three main ways for hepatocytes to acquire FA: transporter-mediated uptake from the blood (173), *de novo* synthesis from acetyl-CoA (174) or receptor-mediated endocytosis of triglyceride-containing chylomicron remnants (173). As free FA are toxic, they are bound to FA-binding proteins in the cytosol (175). The FA can then either be stored in lipid droplets after esterification with glycerol or cholesterol (176), utilized for synthesis of other lipid species (*e.g*., phospholipids) (177) or used as an energy source for β-oxidation in mitochondria or peroxisomes (178). Figure 6 shows the FA metabolism of hepatocytes in a highly simplified manner. Stored triglycerides can be secreted by hepatocytes as very low–density lipoprotein (VLDL) particles together with various phospholipids, cholesterol species, and apolipoproteins (179). VLDL is secreted from hepatocytes to supply other tissues with triglycerides and is regulated by insulin (180).

For us, discovery of the mARC1 p.A165T variant’s association with NAFLD was very surprising, as our group had previously expressed a great variety of common mARC1 and mARC2 variants recombinantly in *E. coli* and studied the variant proteins by *in vitro* assays. In these experiments, Mo loading and *N*-reductive activity of the variant protein was not different between the WT and p.A165T variant proteins (67, 181). Although some authors have predicted the A165T amino acid exchange to drastically impact the structure of mARC1 using *in silico* methods (182), our group has crystallized both WT (129) and p.A165T-mARC1 (183) and observed no structural differences. Also, carriers of the mARC1 p.A165T variant seem to have unaltered mARC1 protein levels (182) or *MTARC1* mRNA levels (159) in their livers. Whether the mARC1 p.A165T variant therefore causes a loss of function or a gain of function is difficult to say. However, some GWAS have shown that the phenotype of the very rare p.R200ter (32) and p.R305ter (162) nonsense variants cause a very similar phenotype as p.A165T, which implies that the variant probably leads to a loss of function. What exact function is lost and how this function is related to mARC’s *N*-reductive activity is unclear at this point.

A very comprehensive study by Lewis *et al.* (163) further confirms the loss-of-function hypothesis, as they correlate predicted *MTARC1* mRNA expression levels with reduced liver fat, total cholesterol, and markers of liver injury. Another study investigating gene expression in cell using induced pluripotent stem cell–derived hepatocytes found differential regulation of *MTARC1* mRNA levels in induced pluripotent stem cell–derived hepatocytes derived from healthy patients and NAFLD patients (184).

There are *in vivo* studies on mice with a liver-specific siRNA-mediated *Mtarc1* knockdown, which have been conducted by pharmaceutical companies seeking to use this strategy as a therapeutical intervention for treatment of NAFLD. Even though each company used different liver-targeted siRNA compounds, results from these studies are comparable (see Table 2) and resemble the phenotype of the mARC1 p.A165T variant observed in humans in the GWAS. An apparent reversal of diet-induced NASH in the murine models indicates that the function of mARC1 in hepatic triglyceride accumulation is probably the same in mice and humans. While liver fat was decreased by liver-specific siRNA-mediated knockdown of *Mtarc1*, there is apparently no decrease in overall body weight, in contrast to *Mtarc2* KO mice (31). This might be a consequence of the liver specificity of the knockdown, which does not affect adipose tissue. On the other hand, no effect on body mass index has been associated with the mARC1 p.A165T variant in humans either (166, 185). One hypothesis could be that mARC1 is responsible for lipid accumulation in the liver, whereas mARC2 mostly contributes to lipid accumulation in adipose tissue.

**Table 2:** Overview of different parameters changed in mice with a liver-specific *Mtarc1* knockdown in mice

Reference	Citation	Strain (diet)	Body weight	Liver weight	Liver TG	Plasma ALT	Plasma TC	Plasma TG
WO2021237097	(193)	B6/C57 (HF/HFr)^a^	→	↓	-	↓	↓	↓
WO2022036126	(145)	c57BL/6 (TD190883)^a^	→	→	↓	↓	↓	→
WO2022183065	(194)	C57BL/6 (Gubra-Amylin NASH)^a^	→	↓	↓	↓	↓	→
Lewis *et al.*	(154)	C57BL/6JRj (D09100310)^a^	↓	↓	↓	→	↑	↑

↓ = lower levels; ↑ = increased levels; → = No change detected.

ALT, alanine transaminase activity; TC, total cholesterol; TG, triglycerides.

a Diets: Hf/HFr, High-fat/High-fructose diet; 60∖xA0kcal% fat, 30%kcal % fructose; TD190883, American Lifestyle-Induced Obesity Syndrome model diet; 22% hydrogenated vegetable oil, 20% sucrose, 12% sucrose, 0.2% cholesterol; Gubra-Amylin NASH, 40% kcal% fat, 20∖xA0kcal % fructose, 2%kcal cholesterol; D09100310, 40% kcal% fat, 20∖xA0kcal % fructose, 2%kcal cholesterol.

According to data provided by the International Mouse Phenotyping Consortium (186), the body weight of *MTARC1* KO animals is not significantly different from that of WT C57BL/6NTac mice over a 16-week period for both male and female mice. This contrasts with the body weight differences observed in *MTARC2* KO animals, where significantly decreased body weight is observed under normal diet and becomes even more pronounced when the animals are fed a high-fat diet (31). Interestingly, according to the Mouse Phenotyping Consortium (186) data, there is also no significant effect on body weight associated with mARC’s known interaction partners CYB5B and NB5R3, but again, these measurements were not done under high-fat diet. Unfortunately, no data examining body weight of *MTARC1* KOmice in comparison to WT animals under high-fat diet is available publicly at the time of writing this review.

Lewis *et al.* furthermore conducted extensive studies on the effect of an siRNA-mediated knockdown of *MTARC1* in cultured primary human hepatocytes. Very interestingly, no effect on the uptake, *de novo* synthesis, or β-oxidation of FAs was reported. Instead, primary hepatocytes seem to secrete larger quantities of triglycerides into the surrounding medium. At the same time, secretion of apolipoprotein B, a major component of VLDL particles, is decreased (163). Several GWAS have shown lower plasma concentrations of apolipoproteins A and B in carriers of the mARC1 p.A165T variant, previously (162, 164, 166). It was therefore hypothesized that decreased mARC1 concentrations lead to secretion of fewer, but larger VLDL particles, resulting in lower intracellular and higher extracellular triglycerides. This is in excellent agreement with GWAS reporting lower plasma cholesterol levels, but increased triglyceride concentrations (see Table 1).

The question of how exactly mARC1 exerts its influence on hepatic triglyceride secretion remains unanswered. Analysis of metabolites in the primary hepatocytes subjected to *MTARC1* knockdown as well as livers from mice with a liver-specific *Mtarc1* knockdown reveal changes in many metabolites connected to phospholipid synthesis (163). Changes in the composition of hepatic phospholipids have been associated with the mARC1 p.A165T variant as well (187, 188). It is not clear whether mARC1 itself influences phospholipid synthesis or if changes to phospholipid synthesis are secondary effects.

Further, it is worth mentioning that variants of several genes other than *MTARC1* have also been associated with the risk of NAFLD and NASH (189, 190). These include patatin-like phospholipase domain-containing 3, transmembrane 6 superfamily member 2 (TM6SF2), and hydroxysteroid 17β-dehydrogenase 13.

mARC1 has in fact been identified as a potential protein–protein interaction partner of TM6SF2 (191). TM6SF2 is reportedly also involved in secretion of lipids from hepatocytes in VLDL particles, by increasing their triglyceride content (192). It is worth mentioning though that the aforementioned study identified interacting proteins after cell lysis (191) and that TM6SF2 is localized in the endoplasmatic reticulum. Thus, the possible interaction between mARC1 and TM6SF might be an artifact or might point toward contact between mitochondria and the endoplasmatic reticulum.

In comparison with other protein targets that influence the prevalence of NAFLD, mARC1 is particularly interesting as, so far, no negative effects have been associated with the protective variant. The increased plasma triglyceride levels and decreased HDL cholesterol appear to have no impact on the risk of cardiovascular disease (32, 166). This is in stark contrast to potential other targets like TM6SF2. Here, the p.E167K loss-of-function variant increases the risk of NAFLD, but paradoxically decreases the risk of cardiovascular diseases (193). Thus, decreasing the risk of NAFLD through targeting of TM6SF2 might come well with increased risk of cardiovascular disease. The currently available data on mARC1 does not indicate such a trade-off.

All in all, mARC1 appears to be a promising target for novel therapies for prevention and/or treatment of NAFLD and NASH. Future research will hopefully reveal, which mARC-catalyzed reaction, if any, is relevant in the pathogenesis of NAFLD. Alternatively, mARC’s involvement in liver disease might be not connected to its function as an oxidoreductase but represent a moonlighting function. Future studies should also clarify the different contributions of mARC1 and mARC2 to lipid metabolism, both in mice and humans, and in different tissues.

## Involvement of mARC proteins in cancer

As mentioned above, mARC1 influences the risk of liver diseases, which might over the course of time progress to hepatocellular carcinoma. However, mARC proteins might also furthermore be involved in certain other types of cancer, for example, colon cancer (194) or bladder cancer (195). It was recently found that *MTARC2* expression might be a favourable indicator in the progression of HCC (196, 197) and the likely mechanism of this effect is particularly interesting: Wu *et al.* (196) propose that mARC2 competes with the tumour suppressor protein p27 for the same degradation pathway. Consequently, increased mARC2 protein levels lead to increased concentrations of p27, which halts cell cycle progression as well as migration of cancer cells (198). We find this mechanism very interesting, as it implies no involvement of mARC2’s Mo centre or catalytic activity of any kind.

## Conclusion

mARC enzymes are the most recently discovered eukaryotic molybdoenzymes. Other than for SUOX or XOR, the physiological function or functions of mARC are not yet fully understood. mARC enzymes can and do catalyze metabolic biotransformation reactions and should be kept in mind when it comes to metabolism of xenobiotics. *N*-reduction can, in some cases, lead to detoxification of harmful *N*-oxygenated compounds. Also, mARC enzymes can be involved in drug metabolism, either by activation of *N*-oxygenated prodrugs or inactivation of drug substances, which require *N*-oxygenated functional groups. The role that eukaryotic Mo enzymes like mARC play in NO homeostasis certainly deserves further investigation.

While it has been shown very conclusively that mARC enzymes play a crucial role in lipid metabolism and diseases associated with lipids, very little is understood about the role of mARC in regulation of lipids. Is mARC directly involved in synthesis of specific lipids? Does mARC synthesise or eliminate some signaling molecules? Or is mARC involved in lipid metabolism by a function unrelated to its enzymatic activity? Are mARC homologs from plants, algae, or bacteria also involved in lipid metabolism in those organisms or is this function specific for animals or mammals in particular?

First phase I clinical trials investigating the tolerability of subcutaneously injected liver-specific *si*RNA–based drugs with healthy volunteers are already being conducted (https://doi.org/10.1016/j.jhep.2023.05.007) and we are excited to see, whether these continue to larger phase II and III trials.

Identifying the exact implication of missense variants in diseases is oftentimes challenging and it could take many more years until we finally understand how mARC and lipid metabolism are connected (199). We are looking forward to future research that will hopefully clarify the function of mARC proteins and MOSC proteins more generally.

## Conflict of interest

The authors declare that they have no conflicts of interest with the contents of this article.
